# The Potential Role of Osteopontin and Furin in Worsening Disease Outcomes in COVID-19 Patients with Pre-Existing Diabetes

**DOI:** 10.3390/cells9112528

**Published:** 2020-11-23

**Authors:** Yvonne Adu-Agyeiwaah, Maria B. Grant, Alexander G. Obukhov

**Affiliations:** 1 Department of Ophthalmology and Visual Sciences, School of Medicine, The University of Alabama at Birmingham, Birmingham, AL 35294, USA; yvonnad@uab.edu (Y.A.-A.); mariagrant@uabmc.edu (M.B.G.); 2 Department of Anatomy, Cell Biology & Physiology, The Indiana University School of Medicine, Indiana University, Indianapolis, IN 46202, USA

**Keywords:** coronavirus, osteopontin, furin, diabetes, ACE2

## Abstract

The novel severe acute respiratory syndrome coronavirus 2 (SARS-CoV-2) has caused the ongoing coronavirus disease 2019 (COVID-19) pandemic, with more than 50 million cases reported globally. Findings have consistently identified an increased severity of SARS-CoV-2 infection in individuals with diabetes. Osteopontin, a cytokine-like matrix-associated phosphoglycoprotein, is elevated in diabetes and drives the expression of furin, a proprotein convertase implicated in the proteolytic processing and activation of several precursors, including chemokines, growth factors, hormones, adhesion molecules, and receptors. Elevated serum furin is a signature of diabetes mellitus progression and is associated with a dysmetabolic phenotype and increased risk of diabetes-linked premature mortality. Additionally, furin plays an important role in enhancing the infectivity of SARS-CoV-2 by promoting its entry and replication in the host cell. Here, we hypothesize that diabetes-induced osteopontin and furin protein upregulation results in worse outcomes in diabetic patients with SARS-CoV-2 infection owing to the roles of these protein in promoting viral infection and increasing metabolic dysfunction. Thus, targeting the osteopontin-furin axis may be a plausible strategy for reducing mortality in SARS-CoV-2 patients with diabetes.

## 1. Introduction

Since December 2019, there has been an outbreak of a novel coronavirus, which has resulted in a global pandemic [1]. The severe acute respiratory syndrome coronavirus 2 (SARS-CoV-2) typically causes pneumonia-like clinical manifestations such as fever, cough, shortness of breath, or difficulty breathing, and even loss of taste or smell [2]. The ongoing coronavirus disease 2019 (COVID-19) pandemic is the third coronavirus-related large-scale pandemic that has occurred over the past two decades. However, the previous pandemics of severe acute respiratory syndrome (SARS) and Middle East respiratory syndrome (MERS) were less brutal than COVID-19 [3,4,5].

**Figure 1:**
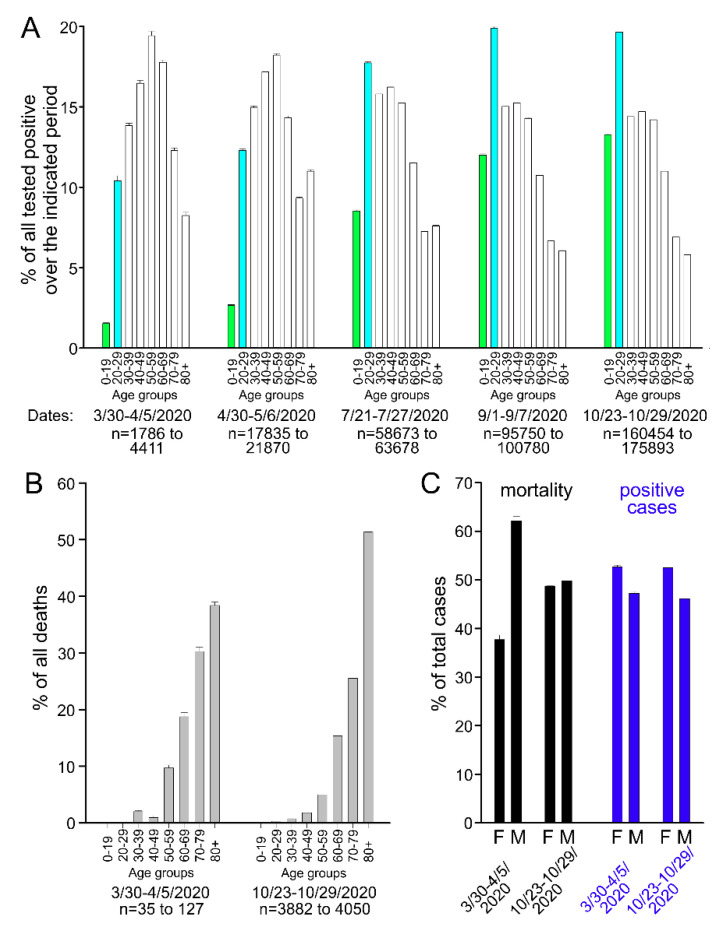
SARS-CoV-2 infection demographics for the state of Indiana from publicly available sources (https://www.regenstrief.org/covid-dashboard/ and https://www.coronavirus.in.gov/). In each case, an average over a seven-day period is provided. (**A**) The number of positive cases during the indicated periods. One Way Repeated Measures Analysis of Variance (*p* < 0.001) for 0–19 and 20–29 age groups. (**B**) Mortality data at the two indicated time periods, which were averaged over a seven-day period. (**C**) Gender demographics for the mortality and positive case data, which were averaged over a seven-day period.

During the initial stages of the current pandemic, only patients at high risk were tested for the SARS-CoV-2 virus, and it first appeared that only people older than 40 were susceptible to COVID-19 [6]. However, with the recent wide-spread testing and back-to-school requirement for students to be tested before attending college, it is apparent that children and young adults are vulnerable to the SARS-CoV-2 virus infection but typically present with mild disease or remain asymptomatic. Indeed, according to the Regenstrief Institute of Indiana (https://www.regenstrief.org/covid-dashboard/) that analyzes the clinical data from the Indiana Network for Patient Care (INPC) managed by the Indiana Health Information Exchange and representing a clinical data repository covering about 100 hospitals and 50,000 providers (publicly available data were pulled on 28 October 2020), only 3.11% of children 0–4 years old (56 out 1800 positive), 0.89% of children and teenagers (153 out of 17,132 positive), and 2.50% young adults and adults (20–29 year old; 731 out of 29,197 positive) were hospitalized. In stark contrast, among the SARS-CoV-2 virus-positive patients, 11.46% of patients of age 50–59 (2539 out of 22,155), 18.91% of patients of 60–69 (3255 out of 17,216), 31.39% of patients of 70–79 (3348 out of 10,667), and 32.13% of patients of 80+ (2937 out of 9142) were hospitalized. Therefore, intensive measures have been directed to protect senior individuals with preexisting health conditions. Figure 1A shows a comparison of statistical data (as of 29 October 2020) publicly available at the website maintained by the state of Indiana (https://www.coronavirus.in.gov/), indicating that the age distribution of individuals infected with SARS-CoV-2 shifted towards the much younger generation in July and August of 2020 and then stabilized at the current level later on. It is likely that the observed change in age distribution of SARS-CoV-2-infected subjects is related to the altered testing pattern of suspected individuals. Additionally, it is possible that the virus has mutated. Indeed, a D614G mutation [7,8,9,10] of SARS-CoV-2, which has been recently identified, is associated with an increased infectivity and faster SARS-CoV-2 virus spread.

Figure 1B shows the data on demographics of positive cases and mortality associated with COVID-19. Individuals older than 50 remain at a greater risk of poor outcome, whereas younger individuals infected with SARS-CoV-2 have a better prognosis. Notably, the gender demographics have also recently changed among SARS-CoV-2-infected individuals who did not survive the disease. Initially, the Indiana state mortality data pulled in March–April 2020 suggested a markedly increased vulnerability of males to die of COVID-19 (Figure 1C). Indeed, recently published evidence also indicated sex differences in SARS-CoV-2 infection, with higher plasma levels of cytokines such as IL-8 and IL-18 and elevated levels of non-classical monocytes (CD14^lo^CD16^+^) in male COVID-19 patients than females. Female COVID-19 patients exhibited a more robust T cell response than male COVID-19 patients. A poor T cell response correlated with a worse clinical course in male COVID-19 patients, whereas higher levels of innate immune cytokines were associated with a worse disease progression in female COVID-19 patients [11]. Nonetheless, more recent data indicate that the gender gap of COVID-19-related deaths decreased in August–September 2020 (SARS-CoV-2-related deaths averaged over a seven-day period ± standard error of mean (SEM): 37.71 ± 0.91% females and 62.14 ± 0.96% males on 30 March 2020 versus 48.74 ± 0.02% females and 49.77 ± 0.02% males on 29 October 2020; Figure 1C). However, the greater susceptibility of females to be infected with SARS-CoV-2 did not change over the same period of time (SARS-CoV-2-positive cases averaged over a seven-day period ± SEM: 52.67 ± 0.36% females and 47.21 ± 0.37% males on 30 March 2020 versus 52.50 ± 0.00% females and 46.10 ± 0.00% males on 29 October 2020; Figure 1C). It is unclear at this time why women present with a higher incidence of SARS-CoV-19 infection. The current Indiana gender distribution ratio is 50.72% females to 49.28% males, according to the 2020 Census (https://worldpopulationreview.com/states/indiana-population). Thus, the number of SARS-CoV-2-positive females is greater than one can expect from the reported gender distribution. It has been recently demonstrated that males exhibit higher plasma soluble angiotensin-converting enzyme 2 (ACE2) protein levels [12,13], and that circulating soluble ACE2 may decrease the ability of SARS-CoV-2 to infect the host cells during COVID-19 infection [14]. It will need to be determined whether the gender-related difference in circulating soluble ACE2 levels contributes to the higher number of infections among females.

## 2. Diabetes and COVID-19

Diabetes Mellitus is a pre-existing condition that results in worse outcomes with COVID-19 infection [15]. It has been identified as an important global health condition that presents a high disease burden, which is further fueled by the global rise in the prevalence of obesity and unhealthy lifestyles [16]. Pre-diabetes and diabetes patients often present with other comorbidities such as dyslipidemia, hypertension, and cardiovascular disease, which further impact the clinical aspects and pathophysiology of COVID-19 in these patients [17]. The complex pathogenesis and pathophysiology of COVID-19 infection is reflected in its multifaceted clinical presentations, which most likely may not be explained by the involvement of just one or two molecular pathways. In diabetes, hyperglycemia and its associated insulin resistance mediate systemic inflammatory response and oxidative stress, which contributes to microvascular pathology by promoting endothelial dysfunction [18,19]. Diabetes can result in multisystem complications with microvascular endpoints including neuropathy, nephropathy, and retinopathy, and it is considered one of the commonest risk factors for developing cardiovascular disease such as ischemic heart disease [16,18]. Studies have identified that complications and comorbidities linked to diabetes are associated with a higher mortality rate in patients subsequently diagnosed with COVID-19 [20]. Obesity is the leading risk factor for developing type 2 diabetes [21], and a higher risk of having severe COVID-19 occurs in the presence of obesity [22].

## 3. Diabetes and Dysregulation of the Renin-Angiotensin System

Potential derangement of several different pathways has been advanced to account for the mechanisms that influence the development of diabetes and its complications. One well researched mechanism involves the renin-angiotensin system (RAS). Many tissues have local RAS expression, including the kidneys, adrenal glands, the heart, the nervous system, and vasculature [23], and the RAS has functions in cardiovascular homeostasis [23,24,25]. The RAS also plays key roles in cellular growth, proliferation, differentiation, migration, and apoptosis, in addition to extracellular matrix remodeling and inflammation [26]. Multiple clinical trials have demonstrated that RAS inhibitors significantly reduce the incidence of vascular complications in Diabetes Mellitus patients [27,28,29,30].

Angiotensin II (Ang II) of RAS has a particularly prominent role in diabetes and has been found to interfere with the insulin-stimulated increase in insulin receptor substrate 1-associated Phosphoinositide 3-kinases activity resulting in insulin resistance [31]. Ang II is implicated in the pathogenesis of hypertension and diabetic microvascular complications such as diabetic retinopathy and diabetic nephropathy [32,33]. Angiotensin-converting enzyme 2 (ACE2) is the primary enzyme of the protective arm of the RAS. ACE2 catalyzes the hydrolysis and conversion of Ang II to angiotensin 1–7 (Ang 1–7), thus countering the activity of Ang II. ACE2 expression is modified by the presence of diabetes in experimental animals and is also low in diabetic individuals. This provides a rationale for mechanisms to regulate ACE2 levels in diabetes.

## 4. The Role of ACE2 in COVID-19

Viruses can take over many cell surface-associated molecules for use as their receptors. The SARS coronaviruses usurp ACE2 for use as their receptors. The entry of the SARS coronaviruses is complex and requires strong receptor binding to ACE2 and proteolytic processing of the SARS spike (S) protein by cellular endopeptidases to allow fusion of the viral and cellular membrane of the host cell [34,35,36,37]. Specifically, the SARS-CoV-2 spike protein has to be cleaved by cell surface host protease TMPRSS2 [38,39] that belongs to the Type II Transmembrane Serine Proteases (TTSPs) family to enable virus–cell membrane fusion involving extensive and irreversible conformational changes [40,41,42]. TMPRSS2 is highly expressed in the heart, liver, prostate, intestines, and lung, implying roles there. Elevated TMPRSS2 activity causes increases in both cell death and viral replication of SARS-CoV in vitro [43] by enhancing priming and subsequently fusion. Remarkably, the activity of TMPRSS2 during SARS-CoV-2 infection can be enhanced by the serine protease, furin [44,45,46].

## 5. Furin and Osteopontin in COVID-19

Furin is a subtilisin-like type I transmembrane serine-protease and proprotein/prohormone convertase from the subtilisin/Kexin (PCSK) family that is expressed in virtually all cells. Human furin is initially produced as a 100-kDa precursor and is rapidly converted into an active ∼94-kDa form [47]. It is one of only three proprotein convertases that possess a transmembrane domain/cytoplasmic tail in the C-terminal region. This feature allows it to cleave its substrates into two distinct intracellular compartments: the trans-Golgi network (TGN) and the endosomal compartment [7]. Furin specifically recognizes the consensus site of Arg-X-Lys/Arg-Arg and is capable of cleaving precursors of a wide variety of proproteins and prohormones [48]. These proteins include growth factors, serum proteins (including proteases of the blood-clotting and complement systems), matrix metalloproteinases, receptors, a disintegrin and metalloproteinase domain-containing protein 17 (ADAM17), interferon-γ, viral-envelope glycoproteins, and bacterial exotoxins. Through proteolytic cleavage of ADAM17, furin also indirectly promotes the ADAM17-dependent activation of tumor necrosis factor, an important proinflammatory cytokine involved in systemic inflammation. Additionally, furin is critical for activating the transmembrane form of the (pro)renin receptor [49], which is implicated in binding and activating prorenin in a non-proteolytic manner to regulate the RAS and maturate the transforming growth factor (TGF)-β1.

**Figure 2:**
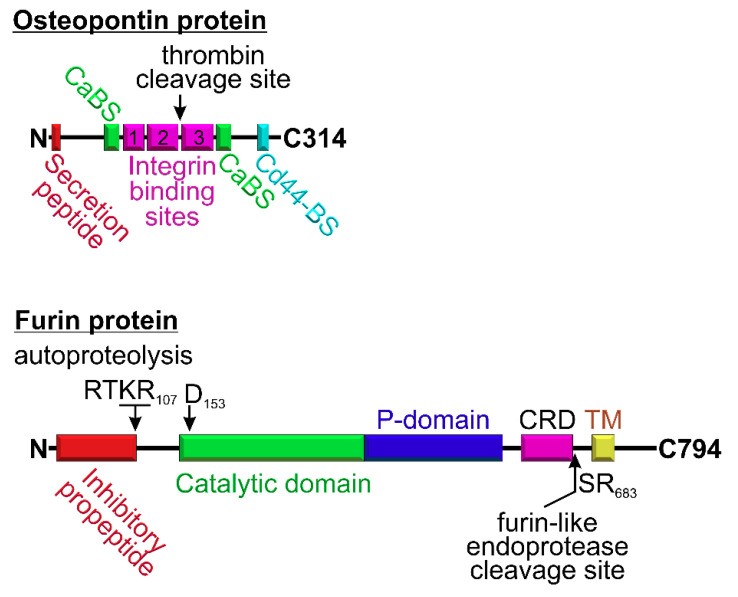
Block diagrams of the osteopontin and furin proteins. Osteopontin contains an N-terminal “secretion peptide” responsible for targeting the protein to the secretory pathways. Osteopontin is subject to thrombin-mediated cleavage, which is critical for exposing the SVVYGLR-integrin binding site. The furin protein is a proprotein convertase that may be membrane bound or shed. The catalytic domain of furin is shown in green. The D153 residue of furin is important for its catalytic activity. In the furin block diagram, there is an N-terminal “inhibitory propeptide” that is blocking the catalytic activity of furin even after autoproteolytic cleavage and needs to be degraded to make furin functionally active; the P-domain stands for the regulatory proprotein convertase domain; TM stands for transmembrane domain; and CRD stands for cysteine-rich domain. The SR683 residues were shown to serve as the proteolytic site for furin shedding.

**Figure 3:**
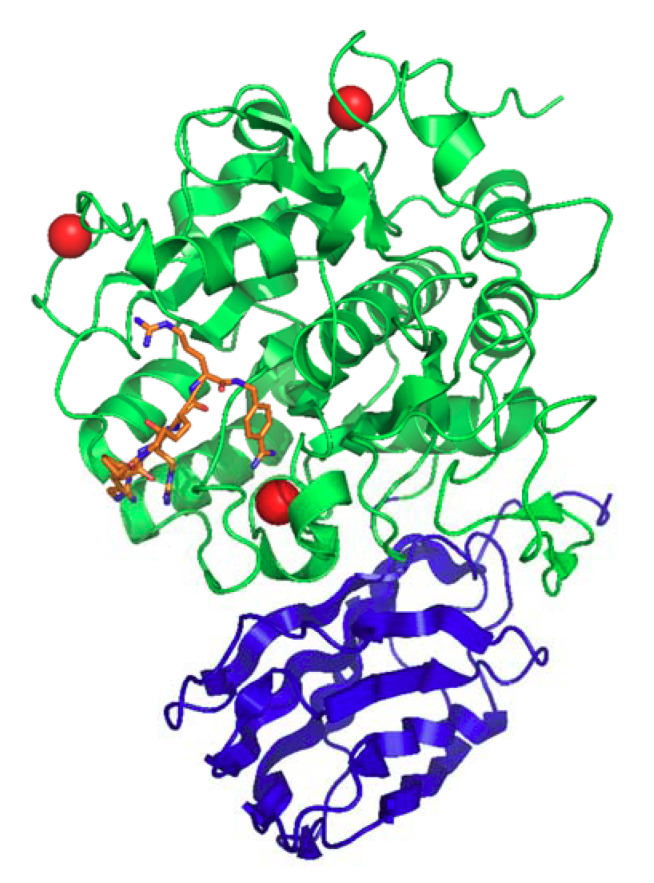
Structure of the soluble domain of human furin protein lacking the N-terminal propeptide (PDB ID: 6 hza). Furin was complexed with an artificial substrate-cyclic model peptide c[glutaryl-Arg-Arg-Lys]-Arg-4-Amba, which is shown as an orange colored stick model with blue nitrogens. Furin is a Ca^2+^-dependent endoprotease. In the structure, the Ca^2+^ cations are shown as red spheres; Na+ cations are shown as blue spheres; and the Cl^-^ anion is shown as a yellow sphere. The furin’s catalytic domain is shown in green, and the P-domain is depicted in blue.

Intracellular furin can be shed into the extracellular milieu [50] when its membrane and C-domains are liberated by other furin-like proprotein convertases or even by autocleavage with furin itself [51]. Notably, the ‘’shed’’ furin retains its catalytic activity. The furin cleavage site, which is important for the protein shedding, was mapped to SR683 amino acid residues [51], the segment located just C-terminally to the cysteine-rich domain (Figure 2). The catalytic activity of furin is regulated by the P-domain (Figure 2 and Figure 3). The rate of catalytically active furin secretion into the blood is enhanced in individuals with diabetes [38,52], suggesting that the protein may contribute to the pathogenesis of diabetes or to SARS-CoV-2 infection in diabetes.

Furin expression is positively regulated by secreted osteopontin, a glycoprotein and proinflammatory cytokine, that is elevated in individuals with diabetes, especially those who exhibit diabetic complications such as diabetic retinopathy and nephropathy [53,54]. Osteopontin belongs to the family of non-collagenous proteins [55] known as SIBLING (small integrin-binding ligand, N-linked glycoprotein) [56], and it was initially isolated from osteoblast and osteocytes [57,58]. Later, osteopontin was found in various cancer cells (reviewed in [59]). Tumor-derived osteopontin functions in tumorigenesis by promoting tumor cell survival and metastasis [60]. Physiologically, osteopontin is secreted by dendritic cells, neutrophils, macrophages, NK cell, T cells, and B cells [61,62,63]. It is also expressed by the kidney epithelial cells and is present in bodily fluids such as milk, blood, and urine [57,64,65]. The osteopontin protein contains three integrin binding sites (Figure 2), namely, the RGD- (**1**), SVVYGLR- (**2**), and ELVTDFTDLPAT- (**3**) binding domains [66]. Additionally, the osteopontin protein has two Ca^2+^ binding sites (CaBS) and a CD44 binding site [67]. Osteopontin is elevated during the inflammation and is secreted by macrophages to serve as chemoattractant for monocytes and T cells and is important for activation, survival, and/or proliferation of macrophages and T cells [67]. Osteopontin is known to promote insulin resistance in diabetes [68].

Osteopontin induces furin expression in a CD44 → p38 MAP kinase → NF-κB-dependent manner [69]. Notably, osteopontin is present in human lung airways [70], the major target tissue of infection in COVID-19 patients. Osteopontin levels are highly upregulated at sites of inflammation [71,72] and in diabetes [73]. Specifically, high glucose was identified as a potent inducer of histone acetylation and methylation causing upregulation of osteopontin gene expression [74]. Additionally, Ang II, the major component of the RAS, was shown to upregulate osteopontin gene expression [75]. Remarkably, it was shown that osteopontin isoforms are present in human and bovine milk and the protein is important during development [76]. Thus, physiological levels of osteopontin are beneficial. However, it remains unclear whether high milk consumption may increase plasma osteopontin levels in individuals with diabetes.

Osteopontin levels are higher in human airways of individuals with mild to moderate asthma, contributing to recruitment of alveolar macrophages [77] and promoting inflammation. Elevated osteopontin secretion was observed in African green monkeys infected with SARS-CoV [78]. Notably, this was associated with developing more severe acute lung injury and acute respiratory distress syndrome.

Osteopontin-regulated furin is broadly involved in maintenance of cellular homeostasis. Higher plasma furin concentration is largely associated with a pronounced dysmetabolic phenotype and an elevated risk of diabetes and premature mortality [38]. Proteolytic cleavage by furin is immensely important in producing a plethora of bioactive proteins and peptides. A study utilizing furin knock-out mice revealed that the genetic ablation of furin resulted in embryonic death at day 11 due to cardiac ventral defects and hemodynamic insufficiency, thus depicting the physiological importance of furin [79]. Likewise, endothelial-cell-specific knock-out of furin resulted in cardiac malformation and death shortly after birth in transgenic mice [80].

Viral pathogens can exploit cellular proteases such as furin during replication and maturation of their preproteins. Furin plays a crucial role in processing and activation of viral glycoproteins [81]. Most viral envelope glycoproteins require proteolytic cleavage to mediate entry into host cells, exploiting cellular endoproteases such as furin for this purpose [82]. Furin-mediated cleavage is important for infection and pathogenicity of numerous evolutionarily diverse virus families, including Herpes-, Corona-, Flavi-, Toga-, Borna-, Bunya-, and Filo, and is implicated in the pathogenesis of the novel β-coronavirus, SARS-CoV-2.

Being ubiquitously expressed, furin is found in several human organs, such as the lungs, heart, and small intestine, that can be invaded by SARS-CoV2. Furin, a pH and Ca^2+^-activated serine protease that is normally housed in the trans-Golgi network (TGN), cleaves the SARS-CoV-2 S protein during virus egress. However, in critically ill COVID-19 patients, the TGN-mediated SARS-CoV-2 egress pathway may be disrupted. In this case, furin “shed” into the blood may mediate the priming of SARS-CoV-2, which may facilitate the virus spread throughout organs of the hosts.

Furin’s ability to promote the maturation of cytokines, such as TNFα, may contribute to the “cytokine storm” in COVID-19 patients with diabetes. Additionally, furin was linked to promoting atherosclerosis and cancer progression. Moreover, SNPs in the furin gene are linked to hypertension. Thus, it is important to investigate the effects of elevated blood plasma furin in the setting of diabetes in COVID-19 subjects and compare the data to those obtained in COVID-19 subjects without diabetes. Such research would also be helpful to better understand the role of furin during atherosclerosis development and cancer progression in COVID-19 patients. Table 1 summarizes recent articles on Furin, Osteopontin, ACE2, diabetes as related to COVID-19.

## 6. Are Osteopontin, Furin, and TMPRSS2 Acting in Concert to Facilitate SARS-CoV-2 Infection?

A growing body of evidence suggests that osteopontin, furin, and TMPRSS2 act in concert for facilitating SARS-CoV-2 infection, with furin playing a presumably precursory role. It has been demonstrated that the cleavage of SARS-CoV-2 spike protein by the serine protease TMPRSS2 is critical for the infectivity of SARS-CoV-2 [44,83,84]. The TMPRSS2 cleavage site is located in a shallow pocket on the lateral surface of the SARS-CoV-2 spike protein [85] that may be obstructed by a protein loop containing an additional canonical cleavage site for furin (PRRAR↓SV) located near of the conserved TMPRSS2 cleavage site. Furin cleavage may increase accessibility of the TMPRSS2 site for TMPRSS2 cleavage in SARS-CoV-2. The SARS-CoV spike protein does not have furin-like proteolytic cleavage sites in the adjacent protein loop. This may at least in part underlie the lower transmissibility and pathogenicity of SARS-CoV compared to SARS-CoV-2. The ability of ‘’shed’’ furin to activate ADAM17 by proteolytic cleavage would result in consequently increased shedding of the soluble ACE2, due to proteolytic cleavage of membrane anchored ACE2, a target of ADAM17. Since ACE2 is the protective axis of the RAS, soluble ACE2 shedding could exacerbate the clinical outcome of COVID-19 patients by reducing ACE2 levels on the surface of the endothelium, promoting vascular dysfunction [85,86]. However, soluble ACE2 may serve to scavenge circulating SARS-CoV-2 and thus serve a protective function [14]. Secreted osteopontin may be elevated in the lungs of COVID-19 patients with diabetes increasing furin expression and shedding, further worsening the COVID-19 patient outcome.

**Figure 4:**
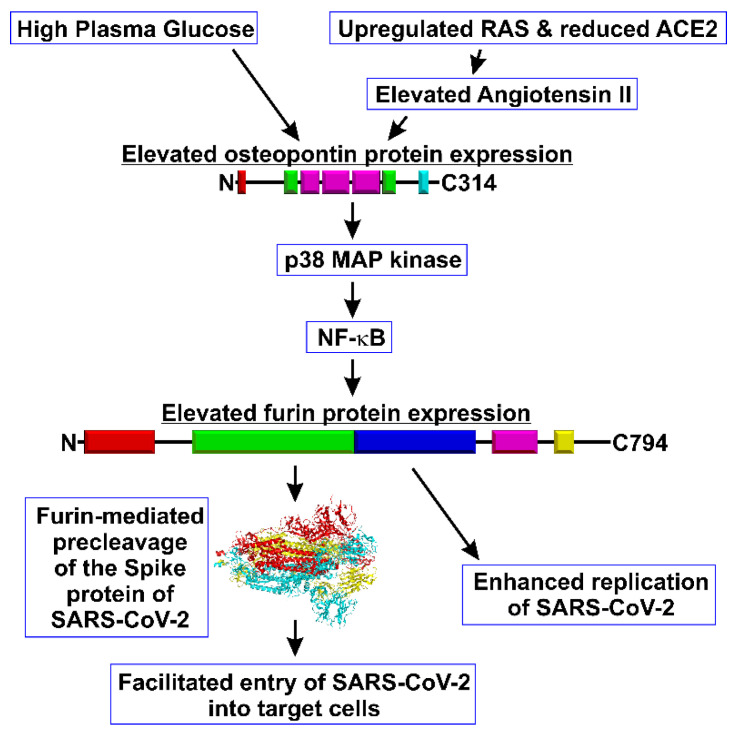
Diagram of the High Glucose–Osteopontin–Furin axis and its putative implication in regulating SARS-CoV-2 infection. Structure of the Spike protein of SARS-CoV-2 was redrawn using the atomic coordinates published in PDB ID: 6VXX.

With this background, we propose a hypothesis that, in COVID-19 patients presenting with diabetes, the overactivity of the “High Glucose-Osteopontin-Furin Axis” enhances the virulence of the SARS-CoV-2 virus by promoting the TMPRSS2-dependent cleavage of the SARS-CoV-2 Spike protein and facilitating SARS-CoV-2 entry into target cells as well as enhancing the replication of SARS-CoV-2 (Figure 4). High Glucose-Osteopontin-driven furin overproduction and subsequent furin shedding may also contribute to reducing the function of the protective axis of RAS by shedding soluble ACE2. Importantly, plasma furin levels are significantly elevated in individuals with diabetes [38,87]; therefore, it may contribute to poor outcomes in COVID-19 patients presenting with diabetes. Remarkably, angiotensin II receptor blockers and statins were shown to reduce elevated plasma levels of osteopontin [88], providing a possible strategy for treatment.

## 7. Future Directions

To test the hypothesis, it is important to ascertain the influence of osteopontin and furin on SARS-CoV-2 virus.

(1) The contribution of TMPRSS2 in regulating the virus infectivity can be investigated utilizing the SARS-CoV-2 isolates harboring the D614G mutation to examine whether the osteopontin-furin axis activation enhances SARS-CoV-2 infectivity.
(2) The effect of increasing levels of osteopontin on furin levels in the supernatants from lung epithelial cell cultures (alveolar type II cells) can be established in the presence or absence of high glucose and/or Ang II.
(3) Bronchoalveolar lavage and blood plasma samples from COVID-19 patients with diabetes can be obtained and analyzed to determine the relative levels of soluble ACE2, osteopontin, and furin present and can be compared to those in COVID-19 patients without diabetes, and healthy subjects. This would provide an estimate of the impact of the High Glucose-Osteopontin-Furin axis to the pathogenesis of COVID-19.
(4) The levels of TMPRSS2, osteopontin, furin, and ACE2 in fixed lung tissue samples from COVID-19 patients with and without diabetes can be assessed.
(5) Inhibitors of p38 MAP kinase and NF-κB signaling as well as statins or Ang II receptor blockers can be employed to assess their protective potential against SARS-CoV-2 infection in a transgenic humanize mouse model expressing human ACE2 in a targeted manner.

## 8. Conclusions

In contemplating the reason for the observed worse phenotype of COVID-19 disease presented by patients with pre-existing diabetes, high levels of ‘’shed’’ furin can be implicated. Here, we proposed that the axis involving High Glucose/Ang II → Osteopontin → p38 MAP kinase → NF-κB → Furin signaling might contribute to the worsening outcome in COVID-19 individuals with diabetes. It is worth noting that the SARS-CoV-2 Spike protein’s susceptibility to proteolytic cleavage by furin may play a key role in SARS-CoV-2 ability to infect the host cells and subsequently invade secondary organs, resulting in worse disease outcomes, including death.

## Figures and Tables

**Table 1:** Summary of recent research on Furin, Osteopontin, diabetes, and angiotensin-converting enzyme 2 (ACE2) as related to corona virus disease 2019 (COVID-19).

Title	Author (Year)	Results/Interpretation
Clinical observation and management of COVID-19 patients	Li et al. (2020) [2]	Patients require respiratory supportive treatment in addition to timely multiorgan evaluation and treatment.
Diabetes is a risk factor for the progression and prognosis of COVID-19	Guo et al. (2020) [20]	Patients with diabetes are at higher risk of severe pneumonia, tissue-injury-related enzymes, and excessive uncontrolled inflammation. This implies that diabetes should be considered a risk factor for rapid progression and poor prognosis in COVID-19 infection.
Risk factors for disease progression in patients with mild to moderate coronavirus disease 2019: a multicenter observational study	Cen et al. (2020) [6]	This study identified aging, male sex, presence of hypertension, diabetes, chronic obstructive pulmonary disease and coronary artery disease as risk factors for disease progression.
A pneumonia outbreak associated with a new coronavirus of probable bat origin	Zhou et al. (2020) [1]	The study characterizes the new coronavirus (SARS-CoV-2) and shows that the virus is 96% identical at the whole genome level to bat coronavirus. It confirms that the virus uses the same cell entry receptor-angiotensin-converting enzyme II (ACE2) as SARS-CoV.
Activation of the SARS-CoV-2 receptor Ace2 by cytokines through pan JAK-STAT enhancers	Hennighausen and Lee (2020) [46]	ACE2 together with TMPRSS2 are important for SARS-CoV-2 host cell entry. Pan JAK-STAT components in mammary alveolar cells and in Type II pneumocytes together with STAT1 and STAT5 autoregulation suggest a role for cytokine-signaling pathways in cells targeted by SARS-CoV-2.
Loss of angiotensin-converting enzyme 2 exacerbates diabetic retinopathy by promoting bone marrow dysfunction	Duan et al. (2018) [33]	ACE2^-/y^-Akita mice have reduction of both short-term and long-term repopulating hematopoietic stem cells, a shift of hematopoiesis towards myelopoiesis, and an impairment of lineage-c-kit+ hematopoietic stem/progenitor cell (HS/PC) migration and proliferation.
Loss of angiotensin-converting enzyme-2 exacerbates diabetic cardiovascular complications and leads to systolic and vascular dysfunction: a critical role of the angiotensin II/AT1 receptor axis	Patel et al. (2012) [32]	Reduction or loss of ACE2 results in increased oxidative stress, excessive extracellular matrix degradation, and vascular dysfunction.
Microglia influence host defense, disease, and repair following murine coronavirus infection of the central nervous system	Mangale et al. (2020) [65]	Elevated expression of disease associated proteins such as Osteopontin, ApoE and Trem2 was found in mice infected with neurotropic coronavirus.
Prognostic significance of serum osteopontin levels in small cell lung cancer	Xu et al. (2020) [70]	Serum osteopontin levels in small cell lung cancer (SCLC) patients were found to be clinically valuable as a biomarker to predict prognosis in SCLC patients.
Tumor-derived osteopontin isoforms cooperate with TRP53 and CCL2 to promote lung metastasis	Giopanou et al. (2016) [60]	Osteopontin modulates cell–cell interactions, thus enhancing tumor metastasis and progression. This study found that intracellular tumor-derived osteopontin promotes tumor cell survival.
Activation of the SARS coronavirus spike protein via sequential proteolytic cleavage at two distinct sites	Belouzard et al. (2009) [40]	Sequential cleavage at two distinct sites in the vicinity of S1/S2 junction of the SARS-CoV Spike protein is required for its full fusion activity.
First comprehensive computational analysis of functional consequences of TMPRSS2 SNPs in susceptibility to SARS-CoV-2 among different populations	Paniri et al. (2020) [39]	The function and structure of TMPRSS2 was affected by 21 SNPs, which influence the protein folding, post translational modifications, splicing, and miRNA effects on the protein.
SARS-CoV-2 cell entry depends on ACE2 and TMPRSS2 and is blocked by a clinically proven protease inhibitor	Hoffmann et al. (2020) [44]	Receptor binding motif analysis revealed that most amino acid residues essential for ACE2 binding by SARS-CoV are conserved in SARS-CoV-2. SARS-CoV-2 uses ACE2 for host cell entry and TMPRSS2, a serine protease, for priming its spike protein. Camostat mesylate, which blocks TMPRSS2 activity, may be useful in inhibiting viral entry into host cells.
Plasma levels of the proprotein convertase furin and incidence of diabetes and mortality	Fernandez et al. (2018) [38]	Individuals with high plasma furin concentration have a pronounced dysmetabolic phenotype and elevated risk of diabetes mellitus and premature mortality.
Furin controls β-cell function via mTORC1 signaling	Brouwers et al. (2020) [52]	β cell dysfunction results from mTORC1-AFT4 hyperactivation in β cells lacking furin.
Significant expression of FURIN and ACE2 on oral epithelial cells may facilitate the efficiency of 2019-nCov entry	Zhong et al. (2020) [37]	Differential expression of ACE2 and Furin was observed on epithelial cells of different oral mucosal tissues, suggesting that tissues of the oral mucosa present a feasible environment for SARS-CoV-2 infection.

## References

[1] Zhou P., Yang X.L., Wang X.G., Hu B., Zhang L., Zhang W., Si H.R., Zhu Y., Li B., Huang C.L. (2020). A pneumonia outbreak associated with a new coronavirus of probable bat origin. Nature.

[2] Li T., Lu H., Zhang W. (2020). Clinical observation and management of COVID-19 patients. Emerg. Microbes Infect..

[3] Drosten C., Gunther S., Preiser W., van der Werf S., Brodt H.R., Becker S., Rabenau H., Panning M., Kolesnikova L., Fouchier R.A. (2003). Identification of a novel coronavirus in patients with severe acute respiratory syndrome. N. Engl. J. Med..

[4] Zaki A.M., van Boheemen S., Bestebroer T.M., Osterhaus A.D., Fouchier R.A. (2012). Isolation of a novel coronavirus from a man with pneumonia in Saudi Arabia. N. Engl. J. Med..

[5] Singh S., Sharma B.B. (2020). Severe acute respiratory syndrome-coronavirus 2 and novel coronavirus disease 2019: An extraordinary pandemic. Lung India.

[6] Cen Y., Chen X., Shen Y., Zhang X.H., Lei Y., Xu C., Jiang W.R., Xu H.T., Chen Y., Zhu J. (2020). Risk factors for disease progression in patients with mild to moderate coronavirus disease 2019-a multi-centre observational study. Clin. Microbiol. Infect..

[7] Chen J., Wang R., Wang M., Wei G.W. (2020). Mutations Strengthened SARS-CoV-2 Infectivity. J. Mol. Biol.

[8] Li Q., Wu J., Nie J., Zhang L., Hao H., Liu S., Zhao C., Zhang Q., Liu H., Nie L. (2020). The Impact of Mutations in SARS-CoV-2 Spike on Viral Infectivity and Antigenicity. Cell.

[9] Korber B., Fischer W.M., Gnanakaran S., Yoon H., Theiler J., Abfalterer W., Hengartner N., Giorgi E.E., Bhattacharya T., Foley B. (2020). Tracking Changes in SARS-CoV-2 Spike: Evidence that D614G Increases Infectivity of the COVID-19 Virus. Cell.

[10] Koyama T., Weeraratne D., Snowdon J.L., Parida L. (2020). Emergence of Drift Variants That May Affect COVID-19 Vaccine Development and Antibody Treatment. Pathogens.

[11] Takahashi T., Ellingson M.K., Wong P., Israelow B., Lucas C., Klein J., Silva J., Mao T., Oh J.E., Tokuyama M. (2020). Sex differences in immune responses that underlie COVID-19 disease outcomes. Nature.

[12] Kornilov S.A., Lucas I., Jade K., Dai C.L., Lovejoy J.C., Magis A.T. (2020). Plasma levels of soluble ACE2are associated with sex, Metabolic Syndrome, and its biomarkers in a large cohort, pointing to a possible mechanism for increased severity in COVID-19. Crit. Care.

[13] Narula S., Yusuf S., Chong M., Ramasundarahettige C., Rangarajan S., Bangdiwala S.I., van Eikels M., Leineweber K., Wu A., Pigeyre M. (2020). Plasma ACE2 and risk of death or cardiometabolic diseases: A case-cohort analysis. Lancet.

[14] Zoufaly A., Poglitsch M., Aberle J.H., Hoepler W., Seitz T., Traugott M., Grieb A., Pawelka E., Laferl H., Wenisch C. (2020). Human recombinant soluble ACE2 in severe COVID-19. Lancet Respir. Med..

[15] Schofield J., Leelarathna L., Thabit H. (2020). COVID-19: Impact of and on Diabetes. Diabetes Ther..

[16] Forouhi N.G., Wareham N.J. (2014). Epidemiology of diabetes. Medicine.

[17] Lumpuy-Castillo J., Lorenzo-Almoros A., Pello-Lazaro A.M., Sanchez-Ferrer C., Egido J., Tunon J., Peiro C., Lorenzo O. (2020). Cardiovascular Damage in COVID-19: Therapeutic Approaches Targeting the Renin-Angiotensin-Aldosterone System. Int. J. Mol. Sci..

[18] Severino P., D’Amato A., Netti L., Pucci M., De Marchis M., Palmirotta R., Volterrani M., Mancone M., Fedele F. (2018). Diabetes Mellitus and Ischemic Heart Disease: The Role of Ion Channels. Int. J. Mol. Sci..

[19] Severino P., D′Amato A., Netti L., Pucci M., Infusino F., Maestrini V., Mancone M., Fedele F. (2019). Myocardial Ischemia and Diabetes Mellitus: Role of Oxidative Stress in the Connection between Cardiac Metabolism and Coronary Blood Flow. J. Diabetes Res..

[20] Guo W., Li M., Dong Y., Zhou H., Zhang Z., Tian C., Qin R., Wang H., Shen Y., Du K. (2020). Diabetes is a risk factor for the progression and prognosis of COVID-19. Diabetes Metab. Res. Rev..

[21] Barnes A.S. (2011). The epidemic of obesity and diabetes: Trends and treatments. Tex. Heart Inst. J..

[22] Gao F., Zheng K.I., Wang X.B., Sun Q.F., Pan K.H., Wang T.Y., Chen Y.P., Targher G., Byrne C.D., George J. (2020). Obesity Is a Risk Factor for Greater COVID-19 Severity. Diabetes Care.

[23] Bader M. (2002). Role of the local renin-angiotensin system in cardiac damage: A minireview focussing on transgenic animal models. J. Mol. Cell. Cardiol..

[24] Obata J.-e., Nakamura T., Takano H., Naito A., Kimura H., Yoshida Y., Shimizu F., Guo D.-F., Inagami T. (2000). Increased gene expression of components of the renin–angiotensin system in glomeruli of genetically hypertensive rats. J. Hypertens..

[25] Bader M. (2010). Tissue renin-angiotensin-aldosterone systems: Targets for pharmacological therapy. Annu Rev. Pharm. Toxicol..

[26] Ribeiro-Oliveira A., Nogueira A.I., Pereira R.M., Boas W.W., Dos Santos R.A., Simoes e Silva A.C. (2008). The renin-angiotensin system and diabetes: An update. Vasc. Health Risk Manag..

[27] Heart Outcomes Prevention Evaluation Study Investigators (2000). Effects of ramipril on cardiovascular and microvascular outcomes in people with diabetes mellitus: Results of the HOPE study and MICRO-HOPE substudy. Lancet.

[28] Brenner B.M., Cooper M.E., de Zeeuw D., Keane W.F., Mitch W.E., Parving H.H., Remuzzi G., Snapinn S.M., Zhang Z., Shahinfar S. (2001). Effects of losartan on renal and cardiovascular outcomes in patients with type 2 diabetes and nephropathy. N. Engl. J. Med..

[29] Jandeleit-Dahm K.A., Tikellis C., Reid C.M., Johnston C.I., Cooper M.E. (2005). Why blockade of the renin-angiotensin system reduces the incidence of new-onset diabetes. J. Hypertens..

[30] Lewis E.J., Hunsicker L.G., Clarke W.R., Berl T., Pohl M.A., Lewis J.B., Ritz E., Atkins R.C., Rohde R., Raz I. (2001). Renoprotective effect of the angiotensin-receptor antagonist irbesartan in patients with nephropathy due to type 2 diabetes. N. Engl. J. Med..

[31] Folli F., Saad M.J., Velloso L., Hansen H., Carandente O., Feener E.P., Kahn C.R. (1999). Crosstalk between insulin and angiotensin II signalling systems. Exp. Clin. Endocrinol. Diabetes.

[32] Patel V.B., Bodiga S., Basu R., Das S.K., Wang W., Wang Z., Lo J., Grant M.B., Zhong J., Kassiri Z. (2012). Loss of angiotensin-converting enzyme-2 exacerbates diabetic cardiovascular complications and leads to systolic and vascular dysfunction: A critical role of the angiotensin II/AT1 receptor axis. Circ. Res..

[33] Duan Y., Beli E., Li Calzi S., Quigley J.L., Miller R.C., Moldovan L., Feng D., Salazar T.E., Hazra S., Al-Sabah J. (2018). Loss of Angiotensin-Converting Enzyme 2 Exacerbates Diabetic Retinopathy by Promoting Bone Marrow Dysfunction. Stem Cells.

[34] Ge X.Y., Li J.L., Yang X.L., Chmura A.A., Zhu G., Epstein J.H., Mazet J.K., Hu B., Zhang W., Peng C. (2013). Isolation and characterization of a bat SARS-like coronavirus that uses the ACE2 receptor. Nature.

[35] Kirchdoerfer R.N., Wang N., Pallesen J., Wrapp D., Turner H.L., Cottrell C.A., Corbett K.S., Graham B.S., McLellan J.S., Ward A.B. (2018). Stabilized coronavirus spikes are resistant to conformational changes induced by receptor recognition or proteolysis. Sci. Rep..

[36] Pallesen J., Wang N., Corbett K.S., Wrapp D., Kirchdoerfer R.N., Turner H.L., Cottrell C.A., Becker M.M., Wang L., Shi W. (2017). Immunogenicity and structures of a rationally designed prefusion MERS-CoV spike antigen. Proc. Natl. Acad. Sci. USA.

[37] Zhong M., Lin B.-P., Gao H.-B., Young A.J., Wang X.-H., Liu C., Wu K.-B., Liu M.-X., Chen J.-M., Huang J.-Y. (2020). Significant expression of FURIN and ACE2 on oral epithelial cells may facilitate the efficiency of SARS-CoV-2 entry. bioRxiv.

[38] Fernandez C., Rysa J., Almgren P., Nilsson J., Engstrom G., Orho-Melander M., Ruskoaho H., Melander O. (2018). Plasma levels of the proprotein convertase furin and incidence of diabetes and mortality. J. Intern. Med..

[39] Paniri A., Hosseini M.M., Akhavan-Niaki H. (2020). First comprehensive computational analysis of functional consequences of TMPRSS2 SNPs in susceptibility to SARS-CoV-2 among different populations. J. Biomol. Struct. Dyn..

[40] Belouzard S., Chu V.C., Whittaker G.R. (2009). Activation of the SARS coronavirus spike protein via sequential proteolytic cleavage at two distinct sites. Proc. Natl. Acad. Sci. USA.

[41] Heald-Sargent T., Gallagher T. (2012). Ready, set, fuse! The coronavirus spike protein and acquisition of fusion competence. Viruses.

[42] Millet J.K., Whittaker G.R. (2015). Host cell proteases: Critical determinants of coronavirus tropism and pathogenesis. Virus Res..

[43] Matsuyama S., Nagata N., Shirato K., Kawase M., Takeda M., Taguchi F. (2010). Efficient activation of the severe acute respiratory syndrome coronavirus spike protein by the transmembrane protease TMPRSS2. J. Virol..

[44] Hoffmann M., Kleine-Weber H., Pohlmann S. (2020). A Multibasic Cleavage Site in the Spike Protein of SARS-CoV-2 Is Essential for Infection of Human Lung Cells. Mol. Cell.

[45] Bestle D., Heindl M.R., Limburg H., Van Lam van T., Pilgram O., Moulton H., Stein D.A., Hardes K., Eickmann M., Dolnik O. (2020). TMPRSS2 and furin are both essential for proteolytic activation of SARS-CoV-2 in human airway cells. Life Sci. Alliance.

[46] Hennighausen L., Lee H.K. (2020). Activation of the SARS-CoV-2 Receptor Ace2 by Cytokines through Pan JAK-STAT Enhancers. SSRN.

[47] Nakayama K. (1997). Furin: A mammalian subtilisin/Kex2p-like endoprotease involved in processing of a wide variety of precursor proteins. Biochem. J..

[48] Molloy S.S., Bresnahan P.A., Leppla S.H., Klimpel K.R., Thomas G. (1992). Human furin is a calcium-dependent serine endoprotease that recognizes the sequence Arg-X-X-Arg and efficiently cleaves anthrax toxin protective antigen. J. Biol. Chem..

[49] Cousin C., Bracquart D., Contrepas A., Corvol P., Muller L., Nguyen G. (2009). Soluble form of the (pro)renin receptor generated by intracellular cleavage by furin is secreted in plasma. Hypertension.

[50] Preininger A., Schlokat U., Mohr G., Himmelspach M., Stichler V., Kyd-Rebenburg A., Plaimauer B., Turecek P.L., Schwarz H.P., Wernhart W. (1999). Strategies for recombinant Furin employment in a biotechnological process: Complete target protein precursor cleavage. Cytotechnology.

[51] Plaimauer B., Mohr G., Wernhart W., Himmelspach M., Dorner F., Schlokat U. (2001). ‘Shed’ furin: Mapping of the cleavage determinants and identification of its C-terminus. Biochem J..

[52] Brouwers B., Coppola I., Vints K., Dislich B., Jouvet N., Van Lommel L., Gounko N.V., Thorrez L., Schuit F., Lichtenthaler S.F. (2020). Furin controls β cell function via mTORC1 signaling. bioRxiv.

[53] Zhang X., Chee W.K., Liu S., Tavintharan S., Sum C.F., Lim S.C., Kumari N. (2018). Association of plasma osteopontin with diabetic retinopathy in Asians with type 2 diabetes. Mol. Vis..

[54] Yamaguchi H., Igarashi M., Hirata A., Tsuchiya H., Sugiyama K., Morita Y., Jimbu Y., Ohnuma H., Daimon M., Tominaga M. (2004). Progression of diabetic nephropathy enhances the plasma osteopontin level in type 2 diabetic patients. Endocr. J..

[55] Mark M.P., Prince C.W., Oosawa T., Gay S., Bronckers A.L., Butler W.T. (1987). Immunohistochemical demonstration of a 44-KD phosphoprotein in developing rat bones. J. Histochem. Cytochem..

[56] Fisher L.W., Torchia D.A., Fohr B., Young M.F., Fedarko N.S. (2001). Flexible structures of SIBLING proteins, bone sialoprotein, and osteopontin. Biochem. Biophys. Res. Commun..

[57] Brown L.F., Berse B., Van de Water L., Papadopoulos-Sergiou A., Perruzzi C.A., Manseau E.J., Dvorak H.F., Senger D.R. (1992). Expression and distribution of osteopontin in human tissues: Widespread association with luminal epithelial surfaces. Mol. Biol. Cell.

[58] Senger D.R., Perruzzi C.A., Papadopoulos A. (1989). Elevated expression of secreted phosphoprotein I (osteopontin, 2ar) as a consequence of neoplastic transformation. Anticancer Res..

[59] Rodrigues L.R., Teixeira J.A., Schmitt F.L., Paulsson M., Lindmark-Mansson H. (2007). The role of osteopontin in tumor progression and metastasis in breast cancer. Cancer Epidemiol. Biomark. Prev..

[60] Giopanou I., Lilis I., Papaleonidopoulos V., Agalioti T., Kanellakis N.I., Spiropoulou N., Spella M., Stathopoulos G.T. (2017). Tumor-derived osteopontin isoforms cooperate with TRP53 and CCL2 to promote lung metastasis. Oncoimmunology.

[61] Haylock D.N., Nilsson S.K. (2006). Osteopontin: A bridge between bone and blood. Br. J. Haematol..

[62] Shinohara M.L., Kim H.J., Kim J.H., Garcia V.A., Cantor H. (2008). Alternative translation of osteopontin generates intracellular and secreted isoforms that mediate distinct biological activities in dendritic cells. Proc. Natl. Acad. Sci. USA.

[63] Zohar R., Suzuki N., Suzuki K., Arora P., Glogauer M., McCulloch C.A., Sodek J. (2000). Intracellular osteopontin is an integral component of the CD44-ERM complex involved in cell migration. J. Cell Physiol..

[64] Lund S.A., Giachelli C.M., Scatena M. (2009). The role of osteopontin in inflammatory processes. J. Cell Commun. Signal..

[65] Mangale V., Syage A.R., Ekiz H.A., Skinner D.D., Cheng Y., Stone C.L., Brown R.M., O’Connell R.M., Green K.N., Lane T.E. (2020). Microglia influence host defense, disease, and repair following murine coronavirus infection of the central nervous system. Glia.

[66] Lok Z.S.Y., Lyle A.N. (2019). Osteopontin in Vascular Disease. Arter. Thromb. Vasc. Biol..

[67] Weber G.F., Ashkar S., Glimcher M.J., Cantor H. (1996). Receptor-ligand interaction between CD44 and osteopontin (Eta-1). Science.

[68] Icer M.A., Gezmen-Karadag M. (2018). The multiple functions and mechanisms of osteopontin. Clin. Biochem..

[69] Kumar V., Behera R., Lohite K., Karnik S., Kundu G.C. (2010). p38 kinase is crucial for osteopontin-induced furin expression that supports cervical cancer progression. Cancer Res..

[70] Xu C., Yuan Q., Wang W., Chi C., Zhang Q., Li L., Yang R., Wang Y. (2020). Prognostic significance of serum osteopontin levels in small cell lung cancer. BMC Pulm. Med..

[71] Liaw L., Birk D.E., Ballas C.B., Whitsitt J.S., Davidson J.M., Hogan B.L. (1998). Altered wound healing in mice lacking a functional osteopontin gene (spp1). J. Clin. Investig..

[72] O’Brien E.R., Garvin M.R., Stewart D.K., Hinohara T., Simpson J.B., Schwartz S.M., Giachelli C.M. (1994). Osteopontin is synthesized by macrophage, smooth muscle, and endothelial cells in primary and restenotic human coronary atherosclerotic plaques. Arter. Thromb..

[73] Gordin D., Forsblom C., Panduru N.M., Thomas M.C., Bjerre M., Soro-Paavonen A., Tolonen N., Sandholm N., Flyvbjerg A., Harjutsalo V. (2014). Osteopontin is a strong predictor of incipient diabetic nephropathy, cardiovascular disease, and all-cause mortality in patients with type 1 diabetes. Diabetes Care.

[74] Cai M., Bompada P., Atac D., Laakso M., Groop L., De Marinis Y. (2016). Epigenetic regulation of glucose-stimulated osteopontin (OPN) expression in diabetic kidney. Biochem. Biophys. Res. Commun..

[75] de Blois D., Lombardi D.M., Su E.J., Clowes A.W., Schwartz S.M., Giachelli C.M. (1996). Angiotensin II induction of osteopontin expression and DNA replication in rat arteries. Hypertension.

[76] Christensen B.S., Sørensen E.S. (2016). Structure, function and nutritional potential of milk osteopontin. Int. Dairy J..

[77] Arjomandi M., Frelinger J., Donde A., Wong H., Yellamilli A., Raymond W. (2011). Secreted osteopontin is highly polymerized in human airways and fragmented in asthmatic airway secretions. PLoS ONE.

[78] Smits S.L., van den Brand J.M., de Lang A., Leijten L.M., van Ijcken W.F., van Amerongen G., Osterhaus A.D., Andeweg A.C., Haagmans B.L. (2011). Distinct severe acute respiratory syndrome coronavirus-induced acute lung injury pathways in two different nonhuman primate species. J. Virol..

[79] Roebroek A.J., Schalken J.A., Leunissen J.A., Onnekink C., Bloemers H.P., Van de Ven W.J. (1986). Evolutionary conserved close linkage of the c-fes/fps proto-oncogene and genetic sequences encoding a receptor-like protein. EMBO J..

[80] Kim W., Essalmani R., Szumska D., Creemers J.W., Roebroek A.J., D’Orleans-Juste P., Bhattacharya S., Seidah N.G., Prat A. (2012). Loss of endothelial furin leads to cardiac malformation and early postnatal death. Mol. Cell Biol..

[81] Jaaks P., Bernasconi M. (2017). The proprotein convertase furin in tumour progression. Int. J. Cancer.

[82] Klenk H.D., Garten W. (1994). Host cell proteases controlling virus pathogenicity. Trends Microbiol..

[83] Hoffmann M., Kleine-Weber H., Schroeder S., Kruger N., Herrler T., Erichsen S., Schiergens T.S., Herrler G., Wu N.H., Nitsche A. (2020). SARS-CoV-2 Cell Entry Depends on ACE2 and TMPRSS2 and Is Blocked by a Clinically Proven Protease Inhibitor. Cell.

[84] Walls A.C., Park Y.J., Tortorici M.A., Wall A., McGuire A.T., Veesler D. (2020). Structure, Function, and Antigenicity of the SARS-CoV-2 Spike Glycoprotein. Cell.

[85] Obukhov A.G., Stevens B.R., Prasad R., Li Calzi S., Boulton M.E., Raizada M.K., Oudit G.Y., Grant M.B. (2020). SARS-CoV-2 Infections and ACE2: Clinical Outcomes Linked With Increased Morbidity and Mortality in Individuals With Diabetes. Diabetes.

[86] Sharma R.K., Stevens B.R., Obukhov A.G., Grant M.B., Oudit G.Y., Li Q., Richards E.M., Pepine C.J., Raizada M.K. (2020). ACE2 (Angiotensin-Converting Enzyme 2) in Cardiopulmonary Diseases: Ramifications for the Control of SARS-CoV-2. Hypertension.

[87] Fathy S.A., Abdel Hamid F.F., Zabut B.M., Jamee A.F., Ali M.A., Abu Mustafa A.M. (2015). Diagnostic utility of BNP, corin and furin as biomarkers for cardiovascular complications in type 2 diabetes mellitus patients. Biomarkers.

[88] Lorenzen J.M., Neunhoffer H., David S., Kielstein J.T., Haller H., Fliser D. (2010). Angiotensin II receptor blocker and statins lower elevated levels of osteopontin in essential hypertension—Results from the EUTOPIA trial. Atherosclerosis.

